# Early administration of lenalidomide after allogeneic hematopoietic stem cell transplantation suppresses graft‐versus‐host disease by inhibiting T‐cell migration to the gastrointestinal tract

**DOI:** 10.1002/iid3.688

**Published:** 2022-08-29

**Authors:** Yukie Tsubokura, Hideaki Yoshimura, Atsushi Satake, Yutaro Nasa, Ryohei Tsuji, Tomoki Ito, Shosaku Nomura

**Affiliations:** ^1^ First Department of Internal Medicine Kansai Medical University Hirakata City Osaka Japan

**Keywords:** graft‐versus‐host disease, hematopoietic stem cell transplantation, lenalidomide, regulatory T cells

## Abstract

**Introduction:**

Allogeneic hematopoietic stem cell transplantation (aHSCT) is a curative treatment for hematopoietic malignancies. Graft‐versus‐host disease (GVHD) is a major complication of aHSCT. After transplantation, the balance of immune conditions, such as proinflammatory cytokine level and T‐cell subset count, influences GVHD magnitude. Lenalidomide (LEN) is an immunomodulatory drug used for treating several hematological malignancies such as multiple myeloma, adult T‐cell lymphoma/leukemia, and follicular lymphoma. However, the impact of LEN on immune responses after aHSCT has not been elucidated.

**Methods:**

We analyzed the lymphocyte composition in naïve mice treated with LEN. Subsequently, we treated host mice with LEN, soon after aHSCT, and analyzed GVHD severity as well as the composition and characteristics of lymphocytes associated with GVHD.

**Results:**

Using a mouse model, we demonstrated the beneficial effects of LEN for treating acute GVHD. Although natural killer cells were slightly increased by LEN, it did not significantly change T‐cell proliferation and the balance of the T‐cell subset in naïve mice. LEN did not modulate the suppressive function of regulatory T cells (Tregs). Unexpectedly, LEN prevented severe GVHD in a mouse acute GVHD model. Donor‐derived lymphocytes were more numerous in host mice treated with LEN than in host mice treated with vehicle. Lymphocyte infiltration of the gastrointestinal tract in host mice treated with LEN was less severe compared to that in host mice treated with vehicle. The percentage of LPAM‐1 (α_4_β_7_‐integrin)‐expressing Foxp3^−^CD4^+^ T cells was significantly lower in host mice treated with LEN than in host mice treated with vehicle, whereas that of LPAM‐1‐expressing Tregs was comparable.

**Conclusions:**

LEN may be useful as a prophylactic agent for acute GVHD‐induced mortality through the inhibition of lymphocyte migration to the gastrointestinal tract. Our data show the effect of LEN on immune responses early after aHSCT and suggest that cereblon, a molecular target of LEN, may be a therapeutic target for preventing acute GVHD‐induced mortality.

## Graphical abstract

Lenalidomide does not interfere with the proliferation of T cells, the development of inducible regulatory T cells and the suppressive function of regulatory T cells. Administration of lenalidomide ameliorates acute graft‐versus‐host disease and is associated with the inhibition of T‐cell migration to the gastrointestinal tract.

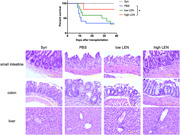

## 1 INTRODUCTION

Allogeneic hematopoietic stem cell transplantation (aHSCT) is a curative therapy for various hematopoietic diseases, especially hematological malignancies. Alloimmune responses mediated by donor‐derived T cells cause the graft‐versus‐leukemia effect, which is an advantage of aHSCT; however, excessive immune responses may cause severe graft‐versus‐host disease (GVHD). Acute GVHD (aGVHD) is a major complication of aHSCT, causing high morbidity and mortality.^1^ Corticosteroids are used as a standard treatment for aGVHD. The response rate of corticosteroid therapy is approximately 50%–60%,^2^, ^3^ and patients who do not respond to corticosteroid therapy have higher mortality than patients who have steroid‐responsive GVHD.^4^, ^5^

Recipients who undergo aHSCT for high‐risk diseases are frequently treated with specific antileukemic agents before and after aHSCT because of a substantial need to prevent or treat disease recurrence. However, some of these drugs, such as tyrosine kinase inhibitors, monoclonal antibodies, and immunomodulatory drugs (IMiDs), may influence immune reactions after aHSCT. For instance, pretransplantation use of anti‐CC chemokine receptor 4 monoclonal antibody is associated with an increased risk of steroid‐resistant aGVHD and worsened clinical outcome in adult T‐cell leukemia/lymphoma patients.^6^ Post‐aHSCT maintenance with sorafenib, a multitargeted tyrosine kinase inhibitor, reduces the risk of relapse and death in patients with FMS‐like tyrosine kinase 3‐internal tandem duplication mutation‐positive acute myeloid leukemia.^7^, ^8^ Sorafenib may induce graft‐versus‐leukemia activity by inducing the production of interleukin‐15 in leukemia cells.^9^ Therefore, it is important to understand the impact of these antileukemic agents on immune responses in aHSCT.

Lenalidomide (LEN) is a derivative of thalidomide, which is infamous for its teratogenicity following use as an antiemetic in pregnant women. LEN shows antitumor activity in some hematological malignancies.^10^, ^11^, ^12^, ^13^ Currently, it is used in Japan for treating patients with multiple myeloma, myelodysplastic syndromes with chromosome 5q deletion, and relapsed/refractory adult T‐cell leukemia/lymphoma. Thalidomide, LEN, and another thalidomide derivative—pomalidomide—are designated as IMiDs because these drugs can increase IL‐2^14^, ^15^, ^16^ and interferon‐γ (IFN‐γ) production in T‐lymphocytes,^17^ decrease proinflammatory cytokine production, and augment innate immunity by enhancing γδ T cell, natural killer (NK) cell, and NK T‐cell activities.^18^, ^19^, ^20^, ^21^ Regarding regulatory T cells (Tregs), the effect of LEN is controversial. Treg counts increased in patients treated with LEN and dexamethasone.^22^ LEN maintenance therapy for multiple myeloma patients increased the percentage of Tregs.^23^ By contrast, some reports showed that LEN may negatively affect the proliferation and suppressive function of Tregs.^24^, ^25^, ^26^ LEN could be used after aHSCT for patients with aggressive adult T‐cell lymphoma/leukemia, relapsed/refractory peripheral T‐cell lymphoma, and multiple myeloma; however, the effect of LEN on alloimmune reaction is unclear. In this study, we investigated the effects of LEN on T and NK cells in syngeneic and allogeneic settings, and its impact on aGVHD. We show that administration of LEN early after aHSCT contributes to protection against severe GVHD through the inhibition of T‐cell migration to the gastrointestinal tract.

## 2 MATERIALS AND METHODS

### 2.1 Mice

Female C57BL/6 (B6) (H‐2^b^) and BALB/c (H‐2^d^) were purchased from Japan SLC. Ly5.1 congenic B6 mice (CD45.1^+^) were purchased from Sankyo Labo Service. B6 (CD45.2^+^) Foxp3 GFP knock‐in (Foxp3.GFP KI) mice were purchased from Jackson Laboratories. CD45.1^+^ Foxp3.GFP KI mice on a B6 background were created by crossing Ly5.1.B6 mice with Foxp3.GFP KI mice. The mice were 8–16 weeks old at the time of sacrifice. All mice were housed in specific pathogen‐free conditions and treated in strict compliance with Animal Facility regulations of the Kansai Medical University. For euthanasia and terminal experiments, they were euthanized either with a CO_2_ inhalant or pentobarbital overdose followed by cervical dislocation. Efforts were made to minimize the suffering of study animals throughout all experiments. All research staff were educated about animal care by the Animal Care Committee of the Kansai Medical University at the beginning of the study. All animal studies were approved by the Animal Care Committee of the Kansai Medical University (approval number. 19‐101).

### 2.2 Antibodies and reagents

Anti‐CD4 (RM4‐5), anti‐CD45.1 (A20), anti‐CD45.2 (104), anti‐CD8α (53‐6.7), anti‐T‐cell receptor‐β (TCRβ) (H57‐597), anti‐CD44 (IM7), anti‐CD62L (MEL‐14), anti‐NK1.1 (PK136), anti‐granzyme B (GB11), anti‐IFN‐γ (XMG1.2), anti‐IL‐17A (TC11‐18H10.1), anti‐IL‐10 (JES5‐16E3), anti‐OX40 (OX‐86), anti‐GITR (DTA‐1), anti‐CD122 (5H4), anti‐ICOS (C398.4A), and anti‐LPAM‐1 (DATK32) antibodies for flow cytometry were purchased from BioLegend. Anti‐Foxp3 (FJK‐16s) was obtained from eBioscience. Fc block (2.4G2) and anti‐CTLA4 antibody (UC10‐4F10‐11) were obtained from Tonbo Bioscience, whereas anti‐IL‐2 antibody (JES6‐5H4) was obtained from Miltenyi Biotec. Aqua fluorescent LIVE/DEAD™ stain and carboxyfluorescein succinimidyl ester (CFSE) was obtained from Invitrogen. Cells were stained using LIVE/DEAD™ stain before surface antibody staining or intracellular staining for IFN‐γ, IL‐17, IL‐2, FoxP3, or CTLA4. LEN was obtained from FUJIFILM Wako Pure Chemical Corporation. A stock solution of LEN was prepared in dimethyl sulfoxide, stored at –80°C, and diluted with sterile phosphate‐buffered saline (PBS) immediately before the experiments were conducted.

### 2.3 Flow cytometry, cell sorting, and data analysis

Flow cytometry was performed using FACSCanto (BD Biosciences). For cell sorting, T cells were purified using the Pan T‐cell Isolation Kit (Miltenyi Biotec) before cell surface staining with an antibody against CD4 and CD8. FoxP3^+^ and Foxp3^−^CD4^+^ T cells were sorted by GFP fluorescence using the Foxp3.GFP KI mouse. Fluorescence‐activated cell sorting (FACS) was performed using the FACSAria cell sorter (BD Biosciences) at the Central Research Laboratory of Kansai Medical University. FACS‐sorted populations were of >95% purity. Data were analyzed using FlowJo software (version 8.8.7; TreeStar). Dead cells were excluded from the analysis using LIVE/DEAD™ fixable aqua dead cell staining. Statistical analysis was performed using Student's *t*‐test or analysis of variance using Prism (GraphPad) as appropriate.

### 2.4 In vitro T‐cell proliferation assays

For Tconv proliferation assays, FACS‐sorted Foxp3.GFP^−^CD4^+^ T cells were labeled with CFSE. CFSE‐labeled Tconvs (5× 10^4^ cells/well) and magnetic‐activated cell sorting (MACS)‐sorted CD11c^+^ dendritic cells (DCs; 1 × 10^5^ cells/well) were cocultured in the presence of anti‐CD3 antibody (0.1 mg/ml) and granulocyte–macrophage colony‐stimulating factor (GM‐CSF) (10 ng/ml) with or without LEN at 37°C and analyzed using flow cytometry after 3 days. CD11c^+^ DCs were obtained from spleens of mice subcutaneously injected 14–21 days prior with FLT3L‐expressing EL4 cells. For Treg proliferation assays, FACS‐sorted Foxp3.GFP^+^CD4^+^ Tregs were labeled with CFSE. CFSE‐labeled Tregs (1 × 10^4^ cells/well) and MACS‐sorted DCs (1 × 10^5^ cells/well) were plated in 200 μl T‐cell media (MEM‐a with 10% fetal bovine serum, 1% penicillin/streptomycin, 10 mM HEPES, and 1 × 10^−5^ M 2‐mercaptoethanol) with mouse GM‐CSF (10 ng/ml; ATGen), human IL‐2 (50 U/ml; PeproTech), and anti‐CD3 (0.1 mg/ml; BD Biosciences) in 96‐well flat‐bottom plates. Cells were cultured with or without LEN at 37°C and analyzed using flow cytometry after 4 days.

### 2.5 In vitro inducible Tregs (iTreg) conversion assay

A total of 5 × 10^4^ FACS‐sorted Tconvs (Foxp3.GFP^−^CD4^+^) and CD8^+^ T cells (Foxp3.GFP^−^CD8^+^) from Foxp3.GFP‐reporter mice were stimulated in 96‐well culture plates with 5 × 10^6^ irradiated splenocytes with anti‐CD3 (2 mg/ml), IL‐2 (50 U/ml), and human tumor growth factor‐β (TGFβ) (0.2 ng/ml for CD4^+^ and 1 ng/ml for CD8^+^ T‐cell cultures). In some wells, LEN was added at different concentrations as indicated. After 5 days, the T cells were analyzed for Foxp3 expression using flow cytometry.

### 2.6 In vivo LEN administration

LEN was dissolved at different concentrations in 400 μl PBS and stored at 4°C for the duration of the experiment. Vehicle (PBS) and LEN were injected intraperitoneally as indicated. Spleen tissue was collected after 1, 2, and 3 weeks of LEN injections (5 consecutive days/week) to evaluate the effect of LEN on T and NK cell counts using flow cytometry.

### 2.7 Treg suppression assays

To determine the suppressive activity of CD4^+^ Tregs, GFP^+^ Tregs were sorted using FACS from the spleens of CD45.1^+^ Foxp3.GFP KI mice. CD4^+^GFP^−^ Tconvs were sorted through FACS from CD45.2^+^Foxp3.GFP KI mice as effectors. Tconvs were CFSE labeled and cocultured with the sorted Tregs at various Tconv:Treg ratios in 200 μl of T‐cell medium and stimulated with 5 × 10^6^ irradiated feeder splenocytes and anti‐CD3 (0.1 mg/ml) in the presence or absence of LEN in 96‐well round‐bottom tissue culture plates. CFSE dilution of Tconvs was analyzed 4 days after coculture, and the division index was calculated using FlowJo software.

### 2.8 Induction and assessment of GVHD

BALB/c mice were administered a single dose of 800 cGy total body irradiation. Irradiated host mice were intravenously injected with 1.0 × 10^6^ T cells enriched using MACS (Miltenyi Biotec) and 5.0 × 10^6^ T‐cell‐depleted bone marrow (BM) cells from allogeneic donor (B6) or syngeneic donor mice. Host mice were treated with vehicle or LEN (2 or 50 mg/kg) for 14 days (0–13 days). After transplantation, mice were monitored every day for survival. Survival curves were plotted using the Kaplan–Meier method and compared using a log‐rank test (*p < .05; GraphPad Prism 6.0). The degree of clinical GVHD was assessed 2–3 times per week until Day 38 using a scoring system that summarizes the changes in five clinical parameters: weight loss, posture, activity, fur texture, and skin integrity.^27^ Mice were humanely euthanized within 12 h of their body weight dropping to <30% of the initial body weight, or when moribund and visual signs of GVHD were present. For histopathological analysis, the small intestine, colon, and liver of recipients were removed and fixed immediately in 10% formalin, embedded in paraffin, sectioned, mounted on slides, and stained with hematoxylin and eosin.

### 2.9 Phenotypic and functional analysis of T cells during GVHD

Irradiated BALB/c mice were injected intravenously with 5 × 10^6^ T‐cell‐depleted BM cells and 1.0 × 10^6^ CD4^+^ T cells from B6 mice (CD45.1^+^). Fourteen days after transplantation, donor cells from the spleen and mesenteric lymph nodes were phenotypically analyzed using flow cytometry. To measure cytokine production, isolated lymphocytes of the spleen and mesenteric lymph nodes were cultured in the presence of phorbol myristate acetate (PMA; 50 ng/ml), ionomycin (1 mg/ml), and brefeldin A (10 mM) for 5.0 and 2.5 h before antibody staining, respectively.

### 2.10 Cytokine analysis

Peripheral blood was collected from host mice 14 days after aHSCT. Cytokine concentrations of serum were analyzed using the BD Cytometric Bead Array system (mouse Th1/Th2/Th17 cytokine kit; BD Biosciences), according to the protocol described by the manufacturer.

### 2.11 Cell isolation from the small intestine

The whole small intestine obtained from host mice 14 days after aHSCT was processed using a gentleMACS Dissociator (Miltenyi Biotec) and mouse lamina propria dissociation kit (Miltenyi Biotec), according to the manufacturer's protocol.

### 2.12 Migration assay of T cells

T cells were magnetically sorted from host mice transplanted from an allogeneic donor. A total of 10^6^ T cells were suspended in 200 μl RPMI and placed in the upper compartment of a 24‐well sterile Transwell device (Corning Life Sciences) that was separated from the bottom well by a 5‐μm filter. The filters of the upper chamber were coated with recombinant mucosal vascular addressin cell adhesion molecule‐1 (MAdCAM‐1; 10 μg/ml; R&D Systems) before incubation. To promote migration, recombinant mouse CCL25 (BioLege) was added to the bottom well (100ng/ml, 1 ml final volume), and incubated at 37°C. After 6 h, the migrated T cells were counted by flow cytometry analysis within the bottom compartment and calculated as the number of T cells that migrated in response to the recombinant mouse MAdCAM‐1.

### 2.13 Statistical analysis

Prism (GraphPad) was used for statistical analysis. Data from independent repeats of experiments were graphed as individual data points with a mean ± SD for each group. The data were graphed and analyzed for statistical significance. The statistical test used to calculate each *p* value is indicated in the figure legends. *p* < .05 were considered significant.

## 3 RESULTS

### 3.1 LEN did not interfere with T‐cell proliferation and homeostasis

**Figure 1:**
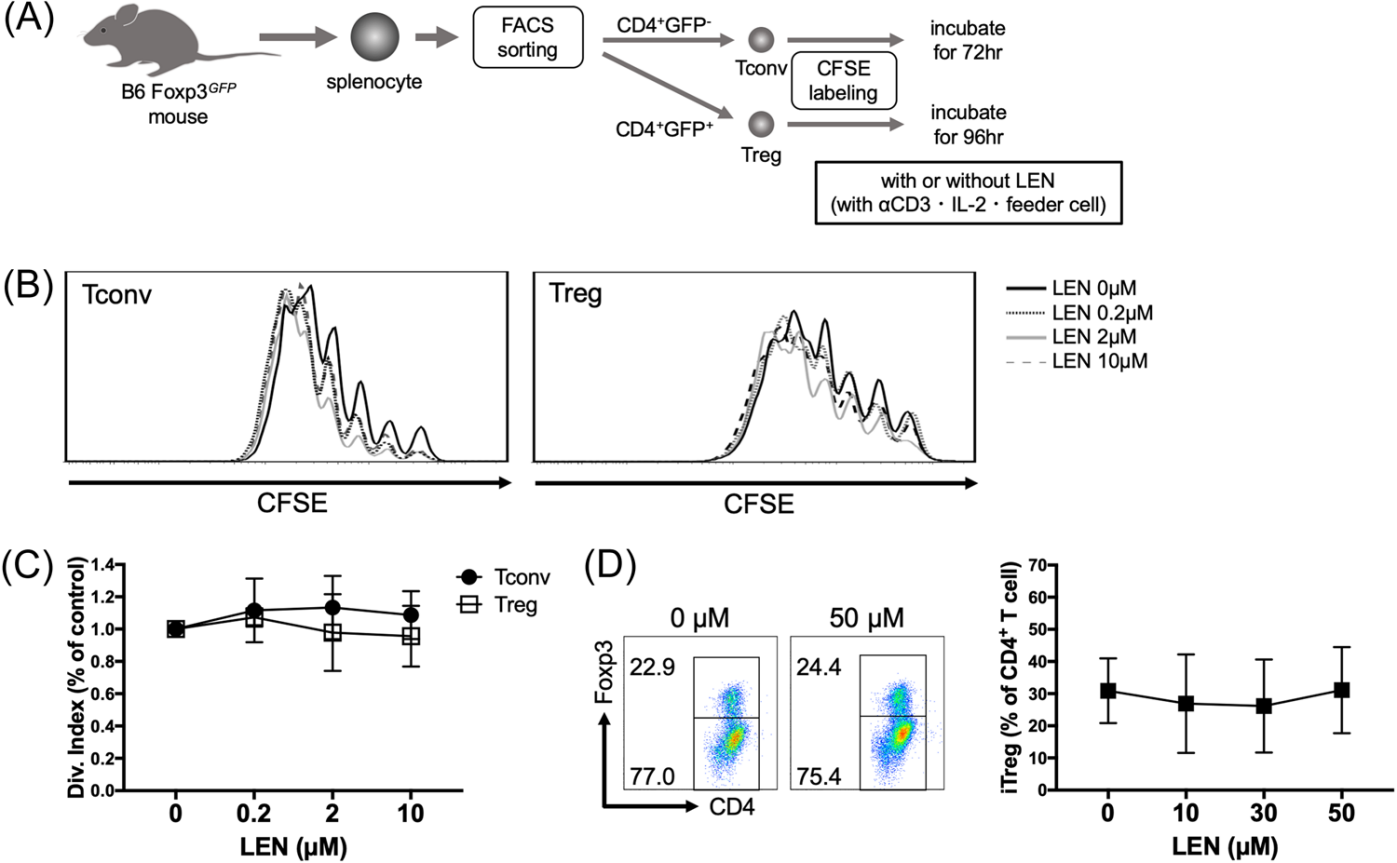
T‐cell proliferation is maintained in the presence of lenalidomide in vitro. (A) Experimental scheme. CFSE‐labeled Tconv and Treg were cultured with syngeneic DCs. (B) Representative histograms showing Tconv and Treg CFSE dilution with several concentrations of LEN. (C) The division index of T cells cultured with LEN was normalized to that of the control, and compiled data from four independent experiments are shown. (D) FACS‐sorted CD4^+^ Foxp3.GFP^−^ Tconvs were stimulated with plate‐bound anti‐CD3/anti‐CD28 and soluble TGFβ and IL‐2 with or without LEN. After 4 days, the cells were harvested and analyzed using flow cytometry. Representative plots (left panel) are shown, and compiled data from three independent experiments are shown as the mean ± SD (right panel). CFSE, carboxyfluorescein succinimidyl ester; FACS, fluorescence‐activated cell sorting; IL‐2, interleukin‐2; LEN, lenalidomide; TGFβ, tumor growth factor‐β; Treg, regulatory T cell.

To investigate the effect of LEN on T cell proliferation, we performed a T‐cell proliferation assay in the presence and absence of LEN. In this analysis, we examined the effect of LEN on Tconv and Treg proliferation in the presence of TCR stimulation (Figure 1A). Tconv proliferation was not inhibited at all by LEN (Figure 1B). Similarly, Treg proliferation was not inhibited by LEN, although inconsiderable inhibition of Treg proliferation was observed at medium and high LEN concentrations (Figure 1C). Next, we examined the effect of LEN on the conversion of iTregs. LEN did not affect iTreg formation stimulated by anti‐CD3 in the presence of TGFβ and IL‐2 (Figure 1D).

**Figure 2:**
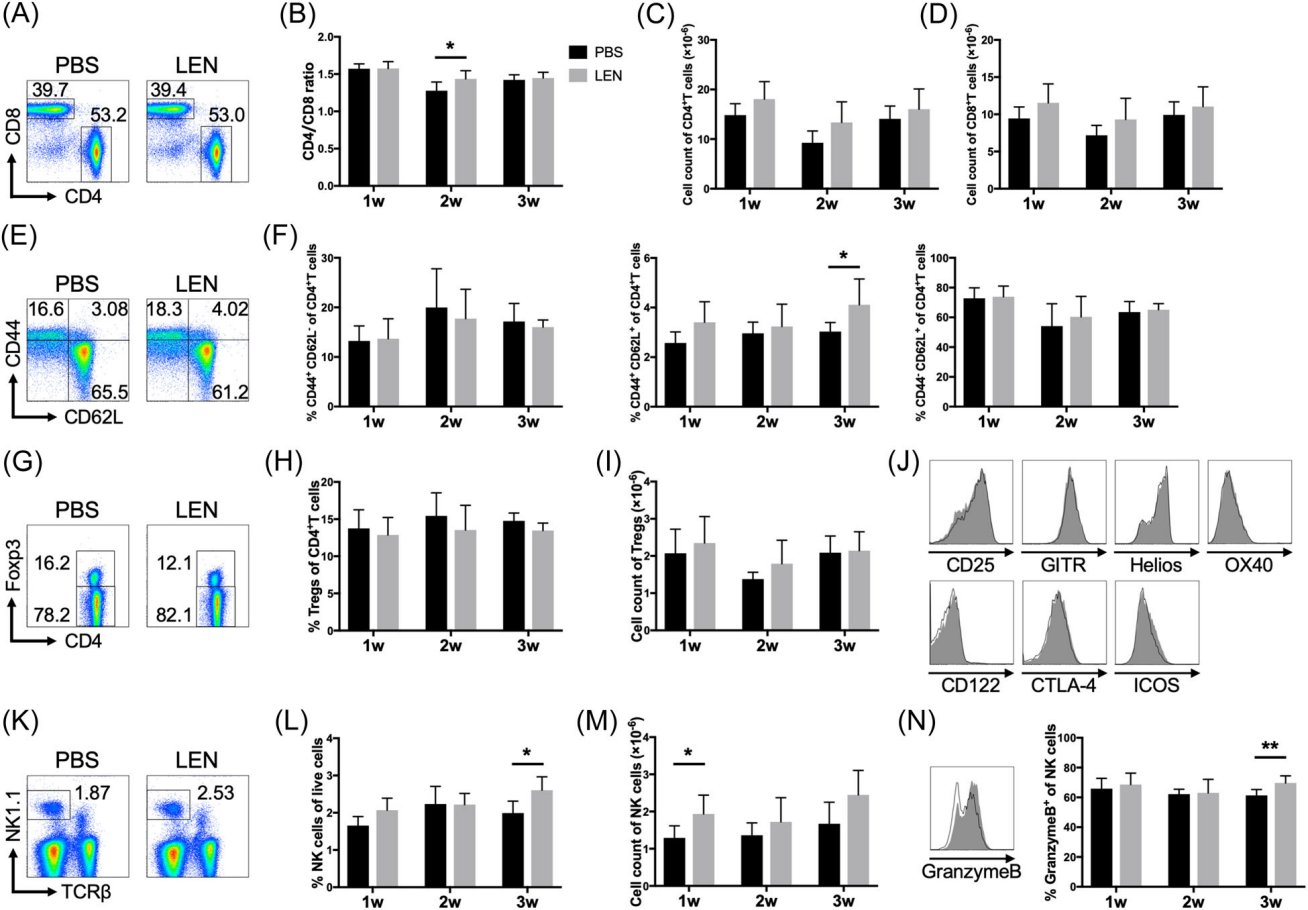
Analysis of T‐cell subset and NK cells in mice injected with or without lenalidomide. B6 mice were injected intraperitoneally with vehicle (PBS) or LEN (50 mg/kg) for 1–3 weeks, and designated cell subsets in the spleen were analyzed using flow cytometry after LEN injection. (A, E, G, K) Representative FACS plots of two independent experiments. (B) CD4^+^/CD8^+^ T‐cell ratio and (C) the absolute number of CD4^+^ and (D) CD8^+^ T cells. (F) The percentage of naïve (CD44^−^CD62L^+^CD4^+^; left), central memory (CD44^+^CD62L^−^; middle), and effector (CD44^+^CD62L^−^) T cells (right). (H) The percentage and (I) absolute number of Tregs. Tregs were analyzed for expression of CD25, GITR, Helios, OX40, CD122, CTLA4, and ICOS. (J) Representative histograms. The shaded and open histograms represent Tregs isolated from B6 mice treated with PBS and LEN, respectively. (L) The percentage and (M) absolute number of NK cells. (N) A representative histogram of granzyme B (left) and the percentage of NK cells expressing granzyme B (right). The shaded and open histograms represent NK cells isolated from B6 mice treated with LEN and PBS for 3 weeks, respectively (left). Data are presented as the mean ± SD of *n* = 6 mice/group from two independent experiments. LEN, lenalidomide; NK, natural killer; PBS, phosphate‐buffered saline. *p < .05 and **p < .01, calculated using the two‐tailed Student's *t*‐test.

Subsequently, to evaluate the effect of LEN in vivo, we injected either vehicle or LEN into wild‐type B6 mice. LEN administration marginally increased T‐cell numbers in the spleen, whereas CD4^+^ T‐cell counts seemed to be slightly increased compared to CD8^+^ T cell counts (Figure 2A–D). In particular, the percentage of central memory T cells (Tcm; CD4^+^CD44^+^CD62^+^) was augmented by LEN administration after 3 weeks (Figure 2E, F). Consistent with the results of in vitro Treg proliferation assay, the percentage and number of Treg in mice injected with LEN were comparable with those injected with the vehicle (Figure 2G–I). Alternatively, NK cell counts were higher in LEN‐injected mice than in vehicle‐injected mice, and granzyme B expression was upregulated after 3 weeks of LEN injection (Figure 2K–N).

### 3.2 LEN did not regulate the suppressive function of Tregs

**Figure 3:**
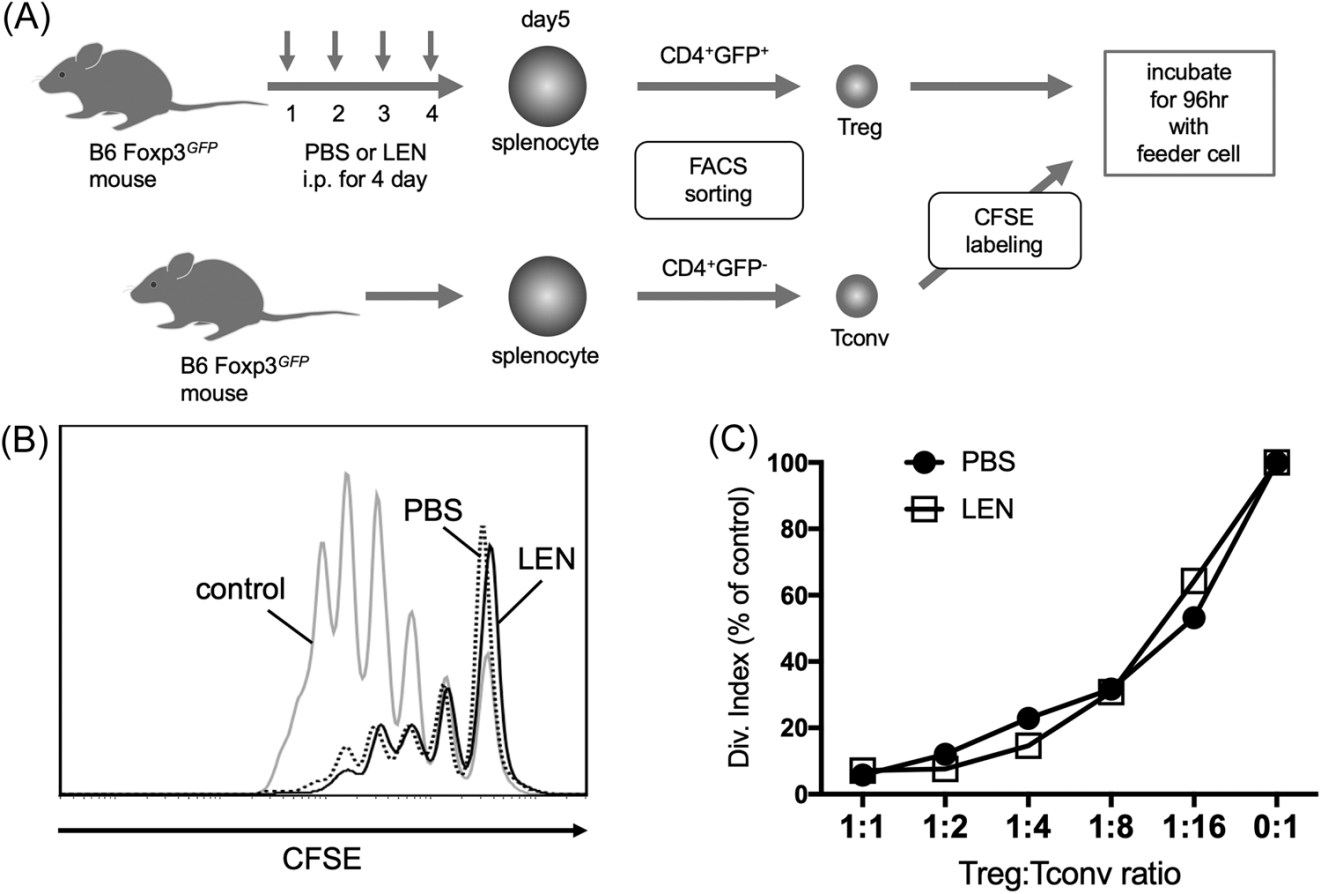
Lenalidomide does not affect the suppressive function of Tregs. (A) B6 Foxp3.GFP KI mice treated with vehicle (PBS) or LEN (10 mg/kg) for 4 days. On Day 5, Tregs isolated from B6 mice treated with PBS and LEN were analyzed for suppressive function. (B) A representative CFSE dilution plot by Tconvs is shown in cultures without Tregs or cultures with a 1:1 T cell:Tconv ratio. (C) The division index was normalized to cultures with no Tregs (0:1 Treg:Tconv ratio) in each experiment. Data are representative of two independent experiments. In each experiment, Tregs were isolated from three mice. CFSE, carboxyfluorescein succinimidyl ester; LEN, lenalidomide; KI, knock‐in; NK, natural killer; PBS, phosphate‐buffered saline; Treg, regulatory T cell.

We analyzed Tregs for the expression of surface markers commonly associated with Treg function. Tregs from LEN‐ and vehicle‐treated mice expressed comparable levels of the high‐affinity IL‐2 receptor (CD122 and CD25) and other coreceptors (OX‐40, GITR, and CTLA‐4) (Figure 2J). Next, we tested the suppressive function of Tregs in the presence and absence of LEN (Figure 3A). Tconv proliferation was equally suppressed by Tregs with or without LEN (Figure 3B, C), suggesting that LEN did not change the suppressive function of Tregs.

### 3.3 High‐dose LEN administration prevented GVHD mortality

**Figure 4:**
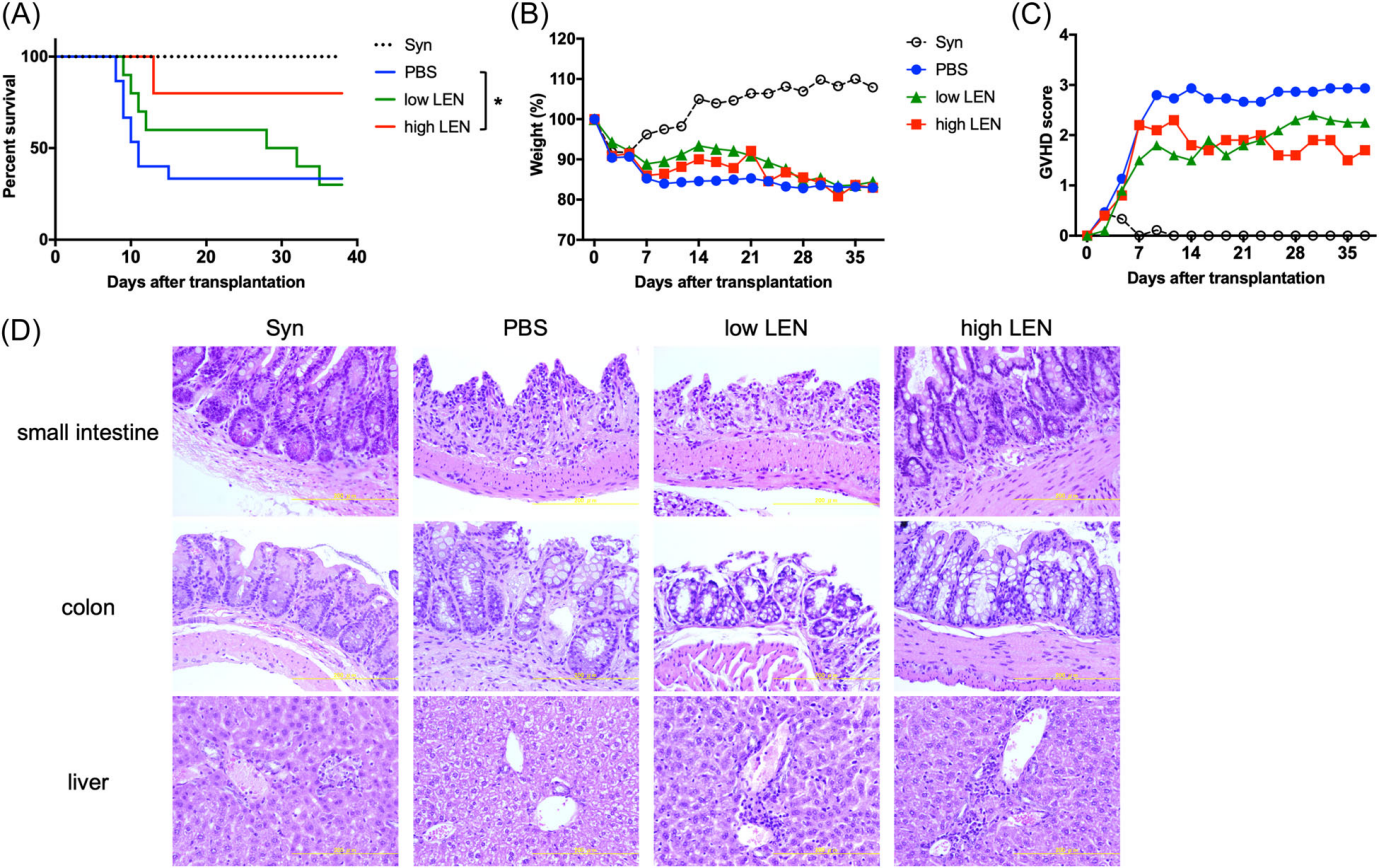
Administration of lenalidomide ameliorates GVHD severity. (A) T cells and T cell‐depleted BM cells from B6 or syngeneic BALB/c mice were injected into lethally irradiated BALB/c mice and subsequently treated with vehicle (PBS), low dose of LEN (2 mg/kg), or high dose of LEN (50 mg/kg) on Days 0–13. Survival was monitored over 38 days. Two independent experiments were combined with *n* = 9 or 10 mice/group to generate the survival curve. *p < .05 using the log‐rank test. (B) Changes in body weight are plotted as the mean of *n* = 9 or 10 mice/group versus days posttransplant. (C) GVHD score are plotted as the mean of n = 9 or 10 mice/group vs. days post transplant. (D) Representative photomicrographs of hematoxylin and eosin‐stained small intestine, colon, and liver sections on Day 14 after transplantation. Scale bar = 200 μm. Images are from a single experiment representative of two independent experiments with three or four mice per experiment. BM, bone marrow; GVHD, graft‐versus‐host disease; LEN, lenalidomide; PBS, phosphate‐buffered saline.

LEN did not significantly affect Tregs and Tconvs in vitro and in a steady state, suggesting that LEN did not change alloimmune responses. To test the effect of LEN on aGVHD, we used a major histocompatibility complex (MHC)‐mismatched aGVHD mouse model. Host mice were treated with vehicle or LEN (2 and 50 mg/kg) for 14 days starting on the day of transplantation, and survival and body weight were monitored. GVHD‐induced mortality in host mice treated with a low dose (2 mg/kg) of LEN was slightly, but not significantly, enhanced compared with that of host mice treated with vehicle (Figure 4A). GVHD‐induced mortality in host mice treated with a high dose (50 mg/kg) of LEN was significantly ameliorated compared with that of host mice treated with vehicle (Figure 4A). Body weight loss and severity of GVHD score of both low‐ and high‐dose LEN‐treated host mice transplanted from an allogeneic donor was marginally mild early after transplantation compared to that of vehicle‐treated mice (Figure 4B, C). Consistent with these clinical findings, the protective effect of a high dose of LEN, such as cellular infiltration and tissue damage, was demonstrated by histopathological analysis of the small intestine and colon (Figure 4D). Alternatively, apparent differences in tissue damage and T‐cell infiltration in the liver were not observed between host mice treated with vehicle and LEN. These results suggested that high‐dose, and not low‐dose, LEN treatment was beneficial for preventing gastrointestinal aGVHD.

### 3.4 LEN preserved donor‐derived lymphocytes in the spleen after aHSCT

**Figure 5:**
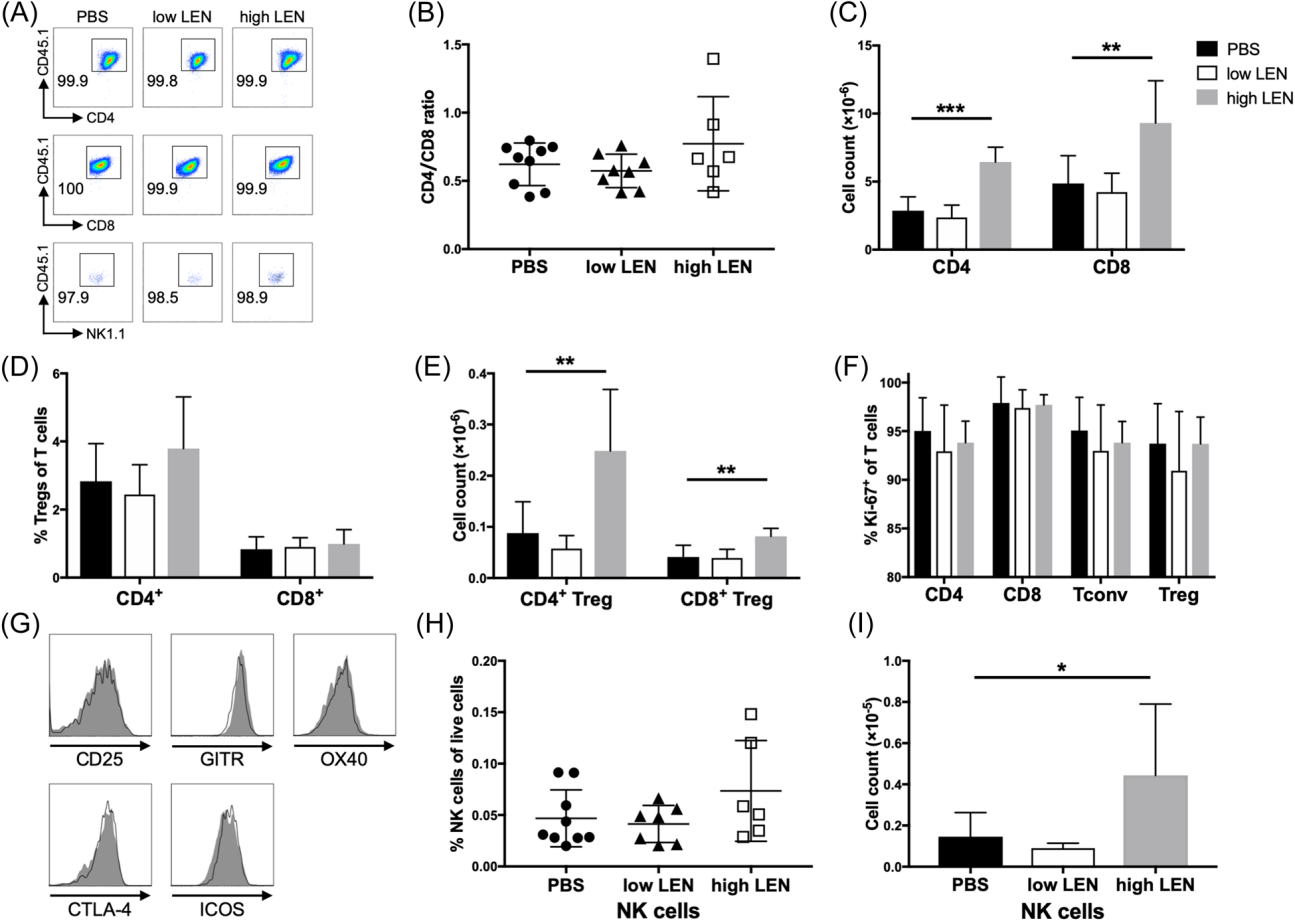
Host mice treated with lenalidomide has a higher number of lymphocytes in the spleen than that in host mice treated with PBS. T cells and T‐cell‐depleted BM cells from B6 or syngeneic BALB/c mice were injected into lethally irradiated BALB/c mice and subsequently treated with vehicle (PBS) and low‐dose or high‐dose LEN on Days 0–13. On Day 14 after transplantation, donor T and NK cells in the spleen were analyzed using flow cytometry. (A) CD45.1^+^ donor chimerism of T (CD4^+^/CD8^+^) and NK (TCRβ^−^NK1.1^+^) cells. (B) CD4^+^/CD8^+^ T‐cell ratio (PBS, circle; low LEN, triangle; high LEN, open square), and (C) the absolute number of CD4^+^ and CD8^+^ T cells. (D) The percentage and (E) absolute number of CD4^+^ and CD8^+^ Tregs. (F) The expression of Ki‐67 in T cells. CD4^+^ Tregs were analyzed for expression of CD25, GITR, OX40, CTLA4, and ICOS. (G) Representative histograms. The shaded and open histograms represent Tregs isolated from host mice treated with LEN and PBS, respectively. (H) The percentage and (I) absolute number of NK cells. Three independent experiments were combined with *n* = 6 (high LEN), 8 (low LEN), or 9 (PBS) mice/group. BM, bone marrow; LEN, lenalidomide; NK, natural killer; PBS, phosphate‐buffered saline; Treg, regulatory T cell. *p < .05, **p < .01, and ***p < .001, calculated by two‐tailed Student's *t*‐test.

Next, to evaluate the effect of LEN on T and NK cells after aHSCT, we examined the number and subset of T cells in host mice on Day 14 after transplantation. Donor T‐cell chimerism in host mice treated with LEN was similar to host mice treated with vehicle (<1% recipient cells) (Figure 5A). Regardless of treatment, CD4^+^ and CD8^+^ T‐cell ratio was almost equal in all host mice (Figure 5B). Both the absolute number of CD4^+^ and CD8^+^ T cells were significantly increased in the spleen after transplantation in host mice treated with a high dose of LEN compared to those in host mice treated with vehicle (PBS vs. high LEN; CD4, *p* < .0001; CD8, *p* < .01), whereas this was not observed in mice treated with a low dose of LEN (Figure 5C). The percentage of Tregs, including CD4^+^ and CD8+ Tregs in host mice treated with low and high doses of LEN, was comparable to that of host mice treated with vehicle (Figure 5D). The absolute number of CD4^+^ and CD8^+^ Tregs significantly increased in host mice treated with a high dose of LEN compared to those treated with vehicle (Figure 5E). The percentage of T cells expressing Ki‐67 was comparable among host mice transplanted from an allogeneic donor regardless of the treatment (Figure 5F). Next, we analyzed the expression of surface markers commonly associated with Tregs in allogeneic host mice. Similar to Tregs isolated from vehicle‐treated host mice, Tregs isolated from host mice treated with high dose LEN expressed components of high‐affinity IL‐2R (CD25 and CD122) and other coreceptors important for the function of Tregs, including OX40, GITR, and CTLA4 (Figure 5G). Additionally, the absolute number of NK cells in host mice treated with a high dose of LEN was higher than that of host mice treated with a vehicle, whereas the percentages of NK cells in host mice treated with low and high doses of LEN were comparable with that of host mice treated with vehicle (Figure 5H, I). These results suggested that administering LEN increased T and NK cells in the spleen while maintaining the balance of the CD4/CD8 ratio and the percentage of NK cells in the allogeneic setting.

### 3.5 Immune polarization of T cells during GVHD was not skewed by LEN

**Figure 6:**
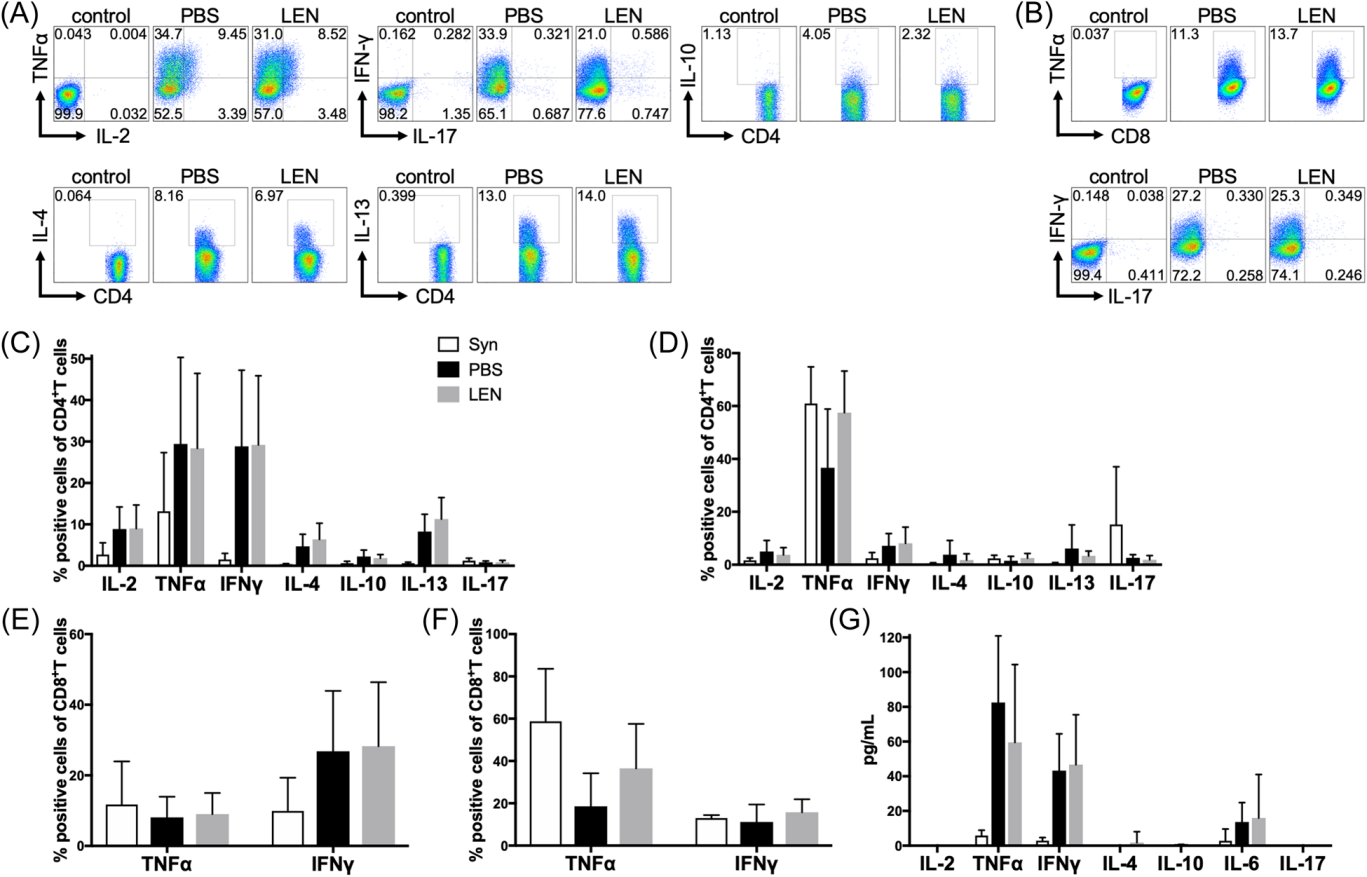
Administration of lenalidomide does not alter the percentage of T cells expressing cytokines after transplantation. T cells and T‐cell‐depleted BM cells from B6 or syngeneic BALB/c mice were injected into lethally irradiated BALB/c mice and subsequently treated with vehicle (PBS) or high dose of LEN on Days 0–13. On Day 14 after transplantation, spleen and mesenteric lymph node cells were stimulated in vitro. The expression of IFN‐γ, IL‐17, and IL‐2 by CD4^+^ T cells (A, C, D) and CD8^+^ T cells (B, E, F) were analyzed using flow cytometry. (A, B) Representative FACS plots of four (spleen) independent experiments. The percentage of IL‐2^+^, TNF‐ α ^+^ IFN‐γ^+^, IL‐4^+^, IL‐10^+^, IL‐13 ^+^, and IL‐17^+^ CD4^+^ T cells in the spleen (C) and mesenteric lymph node (D). The percentage of TNF‐ α^+^, INF‐γ^+^, and IL‐17^+^ CD8^+^ T cells in the spleen (E) and mesenteric lymph node (F). Compiled data four independent experiments are represented as the mean ± SD of *n* = 4 (syngeneic), 13 (LEN), and 17 (PBS). Compiled data from two independent experiments are shown as the mean ± SD of *n* = 4 (syngeneic), 6 (PBS), and 8 (LEN) mice/group. (G) Cytokine concentrations of serum obtained 14 days after aHSCT were analyzed. The levels of IL‐2, TNF‐ α, IFN‐γ, IL‐4, IL‐10, IL‐6, and IL‐17. Compiled data from four independent experiments are shown as the mean ± SD of *n* = 8 (syngeneic), 17 (PBS), and 13 (LEN) mice/group. Statistical analysis was calculated by two‐tailed Student's *t*‐test. aHSCT, allogeneic hematopoietic stem cell transplantation; IL, interleukin; IFN, interferon; LEN, lenalidomide; NK, natural killer; PBS, phosphate‐buffered saline; TNF, tumor necrosis factor.

To gauge the effect of LEN on the production of several cytokines from donor‐derived T cells during GVHD, we examined the percentage of T cells expressing IFN‐γ, IL‐17, IL‐4, IL‐13, IL‐2, IL‐10, and TNF‐α in host mice treated with a high dose of LEN and host mice treated with vehicle, as these cytokines are important for aGVHD pathogenesis and Treg proliferation.^28^, ^29^ Lymphocytes isolated from the spleen were harvested on Day 14 after transplantation and analyzed after stimulation with PMA/ionomycin. The frequencies of CD4^+^ T cells expressing these cytokines in host mice treated with LEN were comparable to those in mice treated with vehicle (Figure 6A, C, E). The frequencies of CD8^+^ T cells expressing these cytokines in host mice treated with a high dose of LEN were comparable to those of host mice treated with vehicle (Figure 6B, D, F). Serum cytokine levels of host mice treated with LEN were also similar to those of host mice treated with vehicle (Figure 6G).

### 3.6 Frequencies of LPAM‐1 integrin‐positive cells were decreased by LEN

**Figure 7:**
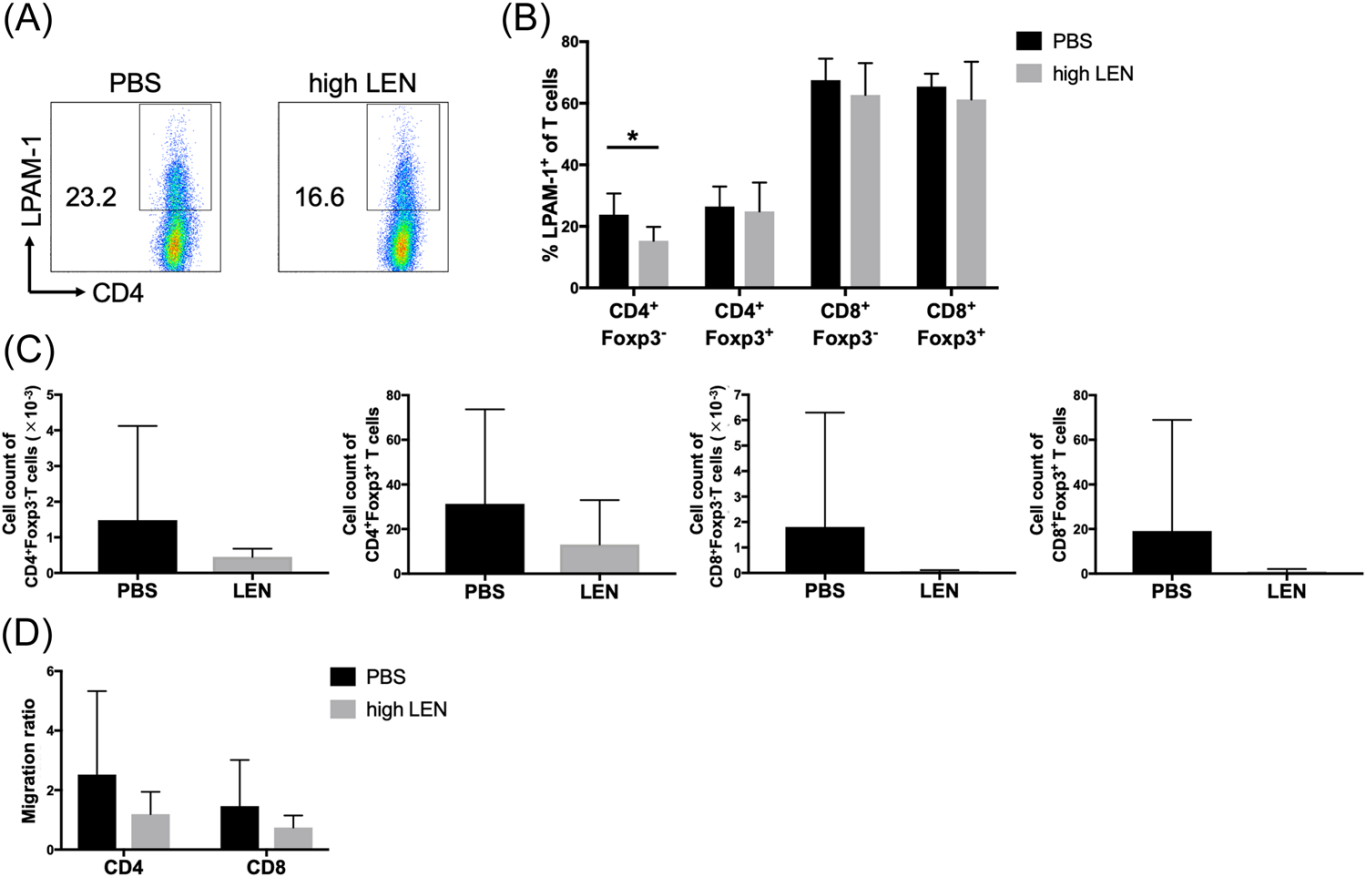
Expression levels of LPAM‐1 on Foxp3^−^CD4^+^ T cells are reduced by administration of lenalidomide. T cells and T‐cell‐depleted BM cells from B6 mice were injected into lethally irradiated BALB/c mice and subsequently treated with vehicle (PBS) and a high dose of LEN on Days 0–13. On Day 14 after transplantation, LPAM‐1 expression on T cells was analyzed. (A) Representative FACS plots of two independent experiments. (B) Percentage of T cells expressing LPAM‐1 is shown. Compiled data from two independent experiments are shown as the mean ± SD of *n* = 5–6 mice/group. (C) Isolated T cells from the small intestine of host mice are shown. Compiled data from three independent experiments are shown as the mean ± SD of n = 7–8 mice/group. (D) MACS‐sorted T cells (1 × 10^6^ cells) were added to the MAdCAM‐1‐coated top chamber of a Transwell device, and CCL25 were added to the bottom wells, following which the top chambers were placed into the wells and incubated at 37°C. After 6 h of incubation, cells were isolated from the bottom well and counted by flow cytometry. Data are expressed as the mean ratio of migration (the number of migrated T cells in the presence of MAdCAM‐1 divided by the number of migrated T cells in the absence of MAdCAM‐1). Compiled data from two independent experiments are shown as the mean ± SD of *n* = 6–7 mice/group. LEN, lenalidomide; MACS, magnetic‐activated cell sorting; MAdCAM‐1, mucosal vascular addressin cell adhesion molecule‐1; PBS, phosphate‐buffered saline; TNF, tumor necrosis factor. *p < .05, calculated by two‐tailed Student's *t*‐test.

Next, we attempted to determine the mechanism by which high‐dose LEN administration induced protection against GVHD. According to the histopathological analysis, the severity of small intestine and colon GVHD was attenuated by a high dose of LEN (Figure 4C). Furthermore, T cells were preserved in the spleen in host mice treated with a high dose of LEN. Thus, we examined whether LEN induces low LPAM‐1 (α4β7‐integrin) expression, a gut‐homing molecule, on T cells after aHSCT. The percentage of LPAM‐1‐positive T cells was significantly lower in Tconv isolated from mice treated with LEN compared to that isolated from mice treated with vehicle (Figure 7A, B). The percentage of Treg‐expressing LPAM‐1 in host mice treated with LEN was comparable to that of mice treated with vehicle (Figure 7B). These results suggest that LEN is associated with Tconv migration inhibition to the gut and may contribute to aGVHD inhibition. Consistent with these results, the number of donor T cells infiltrated in the small intestine isolated from host mice treated with LEN tended to be smaller than that of host mice treated with vehicle (Figure 7C).

As shown in the migration assay evaluating T‐cell migration upon exposure to MAdCAM‐1, which is expressed by venules in the mucosa‐associated lymphoid tissues and can direct the migration of T cells expressing integrin α4β7 into intestinal mucosa, the number of migrated T cells isolated from host mice treated with LEN was higher, but not significantly, than from host mice treated with vehicle (PBS vs. high LEN; CD4, *p* = .25; CD8, *p* = .29) (Figure 7D).

## 4 DISCUSSION

In this study, we examined the effect of LEN on T‐cell immune responses in the presence and absence of alloantigen stimulation. LEN does not interfere with the T‐cell proliferation, the development of iTregs, and the suppressive function of Tregs in the syngeneic and allogeneic settings. We demonstrated that LEN ameliorated aGVHD and was associated with T‐cell migration inhibition to the gastrointestinal tract.

LEN downregulated LPAM‐1 expression after aHSCT. Murai et al.^30^ demonstrated that LPAM‐1 is involved in the induction of acute GVH reaction. LPAM‐1–MAdCAM‐1 interaction was required for the infiltration of donor T cells into the subepithelial dome. LPAM‐1 expression on donor T cells is associated with homing to the gastrointestinal tract and is important in developing intestinal GVHD.^31^, ^32^, ^33^ Additionally, donor T cells imprinted with LPAM‐1 perpetuate severe aGVHD.^34^ Selective anti‐α4β70‐integrin monoclonal antibody (vedolizumab) may have the potential to ameliorate steroid‐resistant gastrointestinal aGVHD.^35^ Consistent with these findings, the frequency of LPAM‐1‐expressing Tconvs was significantly lower in mice treated with a high dose of LEN than in control mice. Additionally, the absolute number of T cells in the spleen was high in host mice treated with LEN, indicating that LEN might preserve T cells in secondary lymphoid organs. Alternatively, we could not find a significant difference between the number of donor‐derived T cells that infiltrated the small intestine of host mice treated with LEN and that in host mice treated with vehicle. This may be because LPAM‐1^+^ T cells could migrate to other gastrointestinal tracts, as well as the small intestine. Although the precise mechanism modulated by LEN to reduce LPAM‐1 expression remains unclear, we believe that the reduced LPAM‐1 expression by high‐dose LEN treatment mainly contributes to GVHD protection because gut GVHD is the main cause of mortality in the mouse model employed by us.

DCs are capable of inducing T‐helper type 1 (Th1) and Th2 responses.^36^ Previous reports showed that IMiDs have immunomodulatory effects on DCs.^37^, ^38^ Recently, our colleagues have reported that IMiDs enhance DC‐mediated Th2 cell responses through upregulated STAT6 and IRF4 expression.^39^ TSLP‐stimulated myeloid DCs enhanced the production of Th2 cytokines due to IMiDs. These modulations of DC‐mediated response may contribute to the amelioration of aGVHD.

Treg is a crucial T‐cell subset that maintains immune tolerance.^40^, ^41^ Tregs suppress excessive autoimmune responses induced by self‐MHC‐reactive T cells. In addition to limiting T‐cell responses against self MHC/peptide complexes and to pathogens, Tregs suppress allogeneic T‐cell responses observed in graft rejection and GVHD.^42^, ^43^, ^44^ The intensity of the alloimmune reaction depends on the balance between Tconvs and Tregs. Thus, the collapse of this balance usually leads to an excessive alloimmune reaction. Thalidomide, LEN, and pomalidomide increase IL‐2 and IFN‐γ production in T‐lymphocytes.^14^, ^15^, ^16^ IL‐2 is a crucial cytokine for Tregs because the maintenance of the Treg population is achieved by a combination of survival and homeostatic proliferation attained by signaling through the cytokine receptor for IL‐2 and TCR^45^ Moreover, IL‐2 is vital and irreplaceable for the development and function of Tregs.^41^ In this context, IMiDs are likely to increase Tregs because Tconvs serve as the main source of IL‐2^28^; however, in this study, we did not observe augmentation of Treg proliferation and function in addition to LEN.

Balance among Th1, Th2, Th17, and Tregs is an important determinant of the severity, manifestation, and tissue distribution of GVHD.^46^ LeBlanc et al.^47^ reported that IMiDs promote T‐cell activation by augmenting CD28 tyrosine phosphorylation on T cells and subsequent nuclear factor‐κB activation, a known downstream target of CD28 signaling. The authors showed that IMiDs promote IFN‐γ secretion of T cells triggered by EBV and influenza. In addition, LEN elevated the concentration of IFN‐γ and IL‐6 in multiple myeloma patients.^48^ Therefore, it was expected that LEN might skew the polarization of T‐cell immune response after aHSCT; however, the percentage of Th1 and Th2 cells was not changed by administering LEN. Th2 cells (IL4^+^ and IL13^+^) marginally increased in host mice treated with LEN.

IMiDs induce expansion and qualitative activation of NK cells and exert an antitumor effect.^19^, ^38^, ^49^, ^50^, ^51^, ^52^ These effects of IMiDs are induced by triggering IL‐2 production by T cells.^51^, ^53^ The effect of LEN on patients who received aHSCT is still unknown. Lioznov et al.^54^ reported that administering LEN after aHSCT for relapsed multiple myeloma induces an increase of activated NK (NKp44^+^) cells and T (HLA‐DR^+^) cells and shows clinical responses. Conversely, Dauguet et al.^55^ reported that LEN downregulates IFN‐γ production and cytotoxicity receptor NKp46 expression. Although the absolute number of NK cells was considerably low because we used purified donor T cells as effector cells for GVHD induction, NK cells expanded due to high‐dose LEN administration in the present study. LEN may contribute to the improvement of transplantation outcomes because NK cells prevent disease relapse, infection, and GVHD.^56^

Some clinical studies with a few patients suggested that administering LEN after aHSCT may induce GVHD.^57^, ^58^, ^59^ Kneppers et al. reported that the LEN maintenance after aHSCT with nonmyeloablative conditioning was not feasible because 14 of 30 patients had to stop LEN due to the development of GVHD.^55^, ^56^, ^57^, ^58^ Sockel et al.^60^ reported that LEN maintenance after aHSCT induced GVHD in patients with myelodysplastic syndrome, in line with data from multiple myeloma patients. In contrast to these results, LEN did not exacerbate GVHD in the present study. Unexpectedly, high‐dose LEN administration significantly mitigated aGVHD. The immunomodulation by IMiDs might have different effects during different posttransplantation phases. LEN was used from the day of transplantation and discontinued 14 days after transplantation in our study, whereas it was initiated after engraftment and continued until disease progression or the emergence of intolerable adverse events in a clinical setting. In addition to the species differences in humans and rodents, this disparity may account for the differential effect of GVHD. Cereblon was identified as the primary target of thalidomide,^61^ and cereblon knockout on myeloma cell lines leads to LEN and pomalidomide resistance.^62^, ^63^ Cereblon is ubiquitously expressed and forms a complex with three other proteins (CUL4, DDB1, and Roc1) to produce the cullin‐4 RING E3 ligase complex, which has E3 ubiquitin ligase activity.^61^, ^64^, ^65^ Ikaros (IKZF1) and Aiolos (IKZF3) were key cereblon‐interacting proteins.^16^, ^66^, ^67^ Cereblon has an enhanced affinity for IKZF1 and IKZF3, with subsequent ubiquitination and degradation of these proteins after binding of an IMiD to cereblon.^16^, ^66^, ^67^ This subsequently leads to changes in gene transcription including decreased expression of IRF4 and increased expression of IL‐2. Although precise mechanisms explaining the discrepancy between the present study and previously reported data are not clear, the results of the present study may provide a clue for using a novel immunotherapy method that targets cereblon for GVHD.

This study has a few limitations. It is unknown whether high‐dose LEN treatment for preventing GVHD is tolerable for patients directly after aHSCT. Next, the effect of LEN in humans may be different from that in rodents. The accurate cause of teratogenicity of thalidomide in humans is not elucidated yet, whereas rodents do not show teratogenicity at all.^61^, ^68^ Although cereblon in humans and rodents is similar, four amino acids of the thalidomide‐binding domain in mice are different from those in humans, and they cause interspecies specificity in antiproliferative effects against myeloma cells.^69^

In conclusion, we have demonstrated that LEN does not affect the proliferation and function of Treg and that it slightly promotes the proliferation of Tcm and NK cells in a steady state. LEN decreased gastrointestinal damage associated with the decrease of LPAM‐1 expression on T cells and mitigates aGVHD by storing donor‐derived T cells in the spleen. Although further investigations are necessary, cereblon, a LEN target, may be a potential therapeutic target for preventing gastrointestinal GVHD.

## AUTHOR CONTRIBUTIONS

Yukie Tsubokura, Hideaki Yoshimura, Yutaro Nasa, and Ryohei Tsuji performed the experiments. Yukie Tsubokura, Hideaki Yoshimura, and Atsushi Satake designed the research and analyzed the data. Tomoki Ito and Shosaku Nomura supervised the laboratory studies. Atsushi Satake supervised the research and wrote the manuscript.

## CONFLICT OF INTEREST

The authors declare no conflict of interest.

## ETHICS STATEMENT

This study was performed in compliance with the protocol reviewed by the Animal Care Committee and was approved by the President of the Kansai Medical University (approval number: 19‐101). The experiments were conducted according to the Rules and Regulations for Animal Experimentation prescribed by the Kansai Medical University.

## Data Availability

The original data for this study are available from the corresponding author on reasonable request.
